# Unraveled roles of hyaluronan in severe COVID-19

**DOI:** 10.17179/excli2020-3215

**Published:** 2021-01-15

**Authors:** Pawared Ontong, Virapong Prachayasittikul

**Affiliations:** 1 Department of Community Medical Technology, Faculty of Medical Technology, Mahidol University, Nakhon Pathom 73170, Thailand; 2 Department of Clinical Microbiology and Applied Technology, Faculty of Medical Technology, Mahidol University, Bangkok 10700, Thailand

**Keywords:** COVID-19, SARS-CoV-2, acute respiratory distress syndrome, hyaluronan, cytokine storm, 4-methylumbelliferone, hyaluronidase

## Abstract

Coronavirus disease 2019 (COVID-19) is a pandemic viral pneumonia caused by severe acute respiratory syndrome coronavirus (SARS-CoV-2). Most of the severe COVID-19 patients come up with trouble breathing, persistent pressure in the chest and developing to acute respiratory distress syndrome (ARDS) with a high mortality rate. Infected lung brings about uncontrolled inflammation followed by the fluid leakage and accumulation of extracellular matrix. Hyaluronan (HA) is an essential component of the extracellular matrix (ECM) and plays crucial roles in both biological and pathological states. It is also primarily located within the respiratory airways and is uprising during COVID-19 infection. Hitherto, the association between COVID-19 pathophysiology and HA is still unclear. Herein, we provide an overview of the pathophysiology of SARS-CoV-2 infection in conjunction with the involvement of HA and the diminution of HA for therapeutic potential of COVID-19. For severe patients, HA depletion may be beneficial for preventing ARDS while monitoring and managing HA level in lung may improve survival rate of patients.

## Introduction

Coronaviruses constitute a group of RNA viruses infecting animals and human which cause mild to severe and critical respiratory syndrome. Since the years of 2002 to 2012, there are two highly pathogenic coronaviruses of zoonotic origin namely severe acute respiratory syndrome coronavirus (SARS-CoV) and Middle East respiratory syndrome coronavirus (MERS-CoV) that correspondingly propel the emergence of coronaviruses as a new public health concern in the twenty-first century (Cui et al., 2019[9]). In December 2019, the first and unusual viral pneumonia case was recorded in Wuhan, China. It was a new strain that has not previously been identified in humans. A novel coronavirus designated as SARS-CoV-2, also known as coronavirus disease 2019 (COVID-19), has spread rapidly all over the world (Wu et al., 2020[37]). SARS-CoV-2 infects people of all ages but those at higher risk of developing severe diseases are the elderly and those with underlying comorbidities. 

Hyaluronan (HA) is a major extracellular matrix (ECM) compound in every vital organ system. It has a crucial role in pulmonary health and disease. Imbalance of HA production and degradation causes respiratory abnormality. Specifically, lethal cases of COVID-19 have shown an accumulation of hyaluronan in alveolar spaces of the lungs which is correlated with the occurrence of hypoxemia and respiratory failure in the critical patient group (Hellman et al., 2020[19]). While the molecular mechanisms on pathogenesis of SARS-CoV-2 infection remain largely unclear. There is a high possibility that HA storming occurs simultaneously during the progression of the diseases. This review will focus on the pathophysiology and the involvement of HA in COVID-19. Potential modality of monitoring HA on the acute respiratory distress syndrome (ARDS) in COVID-19 is also described.

## COVID-19 Pathophysiology

The pathophysiology of SARS-CoV-2 infection closely resembles that of SARS-CoV infection in which the aggressive inflammatory responses are strongly implicated in the resulting damage of airways. Uncontrolled inflammation imposes multi-organ failure, especially of the cardiac, hepatic and renal systems. Most of the patients with SARS-CoV infection progressed to renal failure while also carrying a high mortality (Chu et al., 2005[7]). An estimated 70 % of severe COVID-19 patients developed to ARDS which is characterized by difficulty in breathing and low blood oxygen level that results in fatality. Moreover, the viral infection stimulates immune response that subsequently generates cytokine storm and sepsis which are the cause of death in about 28 % of fetal COVID-19 cases (Tay et al., 2020[33]). Possible key mechanisms that may play a role in the pathophysiology of multi-organ injury as a secondary consequence of SARS-CoV-2 infection include direct viral toxicity, dysregulation of the renin-angiotensin-aldosterone system (RAAS), induction of cytokine storm, and endothelial dysfunction.

SARS-CoV-2 is transmitted primarily via respiratory droplets and direct contact while airborne transmission can possibly occur under special circumstances. SARS-CoV2 binds to the host cells through the angiotensin-converting enzyme 2 (ACE2) expressing cells which are located in the lung such as epithelial cells, alveolar epithelial cells, vascular endothelial cells, fibroblasts and macrophages (Yuki et al., 2020[40]). Cell entry process requires priming of the spike protein by the transmembrane protease, serine 2 (TMPRSS2) or other proteases. The co-expression on cell surfaces of ACE2 and TMPRSS2 is required for the completion of this entry process. Once viruses bind to host receptors, they enter the host cells through endocytosis or membrane fusion. This is followed by the release of viral contents inside the host cells whereby the viral RNA enters the nucleus for replication. Particularly, the viral mRNA encodes the biosynthesis of viral proteins. Afterward, new viral particles are made and released (Yuki et al., 2020[40]). Active replication and release of the virus cause host cells to undergo the highly proinflammatory death program such as necrosis, pyroptosis and necroptosis. The lytic death cells release damage-associated molecular patterns (DAMPs^1^), pathogen-associated molecular patterns (PAMPs) and high mobility group box 1 protein (HMGB1^2^). HMGB-1-DAMP/ PAMP complexes consequently bind to the receptor for advanced glycation end product (RAGE^3^), abundantly expressed in the lungs where the complexes are endocytosed, and subsequently activates the proinflammatory cytokine cascade, induces pyroptosis and initiates coagulation (Andersson et al., 2020[1]). 

The entry of SARS-CoV2 causes the downregulation of ACE2, which brings about the dysregulation of RAAS (Bourgonje et al., 2020[5]). Particularly, RAAS is a hormonal system that regulates key physiological processes of the body including blood pressure, fluid and electrolyte balance, systemic vascular resistance, and tissue growth. ACE2 is a counter-regulator for RAAS, which functions for the cleavages of angiotensin I (Ang I) into inactive Ang 1-9 as well as angiotensin II (Ang II) into Ang 1-7 which is a vasodilator agent that plays an important role in cardiovascular organs, such as heart, blood vessels, and kidneys. Moreover, Ang 1-7 has been shown to attenuate inflammation and improve lung function in ARDS model (Wosten-van Asperen et al., 2011[36]). RAAS over-activation could cause the accumulation of Ang II followed by the lack of Ang 1-7, which increased lung damage and edema. Many cases of SARS-CoV2 infection have been reported on the manifestation of acute respiratory distress syndrome (Hanff et al., 2020[18]), cardiac injury (Bonow et al., 2020[4]), neurologic involvement (Mao et al., 2020[24]), and gastrointestinal distress (Han et al., 2020[17]).

## Immune Response Induced Cytokine Storm

As virus enters the cell, it not only causes lytic cell death but also induces vascular leakage by releasing the proinflammatory cytokines. This is followed by the activation of the host immune response which is often seen in the distal airway. There are three main components of the innate immune response including epithelial cells, alveolar macrophages and dendritic cells (DCs). At early stage, the infected cells release inflammatory cytokines and chemokines such as IL-1β, IL-6, IL-10, IL-18, tumor necrosis factor (TNF-α), macrophage inflammatory protein 1α (MIP1α), MIP1β, and monocyte chemoattractant protein-1 (MCP-1) (Sun et al., 2020[32]; Tay et al., 2020[33]). In addition, SARS-CoV2 infected lung epithelial cells produce IL-8 which is a chemoattractant for neutrophils and T-cells. Particularly, neutrophil degranulation leads to the release of proteases, reactive oxygen and nitrogen species, leukotriene, and platelet activating factor (PAF). Alveolar macrophages and DCs, which are located under the epithelium, serve as an innate mechanism to phagocytose infected apoptotic cells and act as an antigen-presenting cell (APC) for the initiation of the adaptive immune responses against the viral infection. Afterward, APCs will present the viral antigen via MHC class II leading to the CD4+ T-cell-inducing B-cell activation, and the release of antibodies targeting the virus, whereas the CD8+ T cells provide cytotoxic activity that destroys infected cells.

## Endothelial Dysfunction

Cytokine storm causes the uncontrolled inflammation that leads to the injury of lung endothelial cells followed by the pulmonary capillaries leakage, thereby leading to interstitial and pulmonary edema. In addition, it also causes endothelialitis followed by the destruction of endothelial glycocalyx (EG). Under physiological condition, the EG serves as a crucial player in conferring vascular integrity such as delimiting the space between the blood and the endothelium as well as controlling vessel permeability. The EG also modulates inflammation and thrombosis by confining the adhesion of platelets and leukocytes. More importantly, the EG responds to blood flow by mechanosensing via the shear stress transduction (Dogne and Flamion, 2020[11]; Dogne et al., 2018[12]). These functions will be dysregulated upon the uncontrolled inflammatory condition and leads to vascular leakage. Furthermore, the inflammation of vascular endothelium will activate the coagulation cascade by decreasing fibrinolysis and thrombin, thereby resulting in microthrombi deposition, microvascular dysfunction, and vasoconstriction (Fraser et al., 2020[15]). All of these processes speed up the neutrophil recruitment and infiltration into the lung leading to the damage of the pneumocyte. The level of gas exchange and surfactant will be depreciated arising from the lack of type I and II pneumocyte along with the dysfunction of microvessels. Both the interstadial and alveolar edema in combination with the alveolar collapse cause the ventilation-perfusion mismatch followed by intrapulmonary shunt, hypoxia, and respiratory failure.

## Physiological Function of HA

HA, also called hyaluronic acid, is a large non-sulfated glycosaminoglycan polymer of ECM comprising of repeating disaccharide units of D-glucuronic acid and N-acetyl-D-glucosamine that are linked via alternating β-1,4 and β-1,3 glyosidic bonds. HA can be found in vertebrate tissues, as a key component of the ECM. In the lungs, it is a crucial component in the basement membrane region of bronchial, bronchiolar epithelium, inter-alveolar/peri-alveolar tissues and endothelial glycocalyx. In addition, HA has been found at the surface of alveolar macrophages and Type II alveolar epithelial cells (Johnson et al., 2018[20]). It is synthesized by three HA synthases (HAS1, HAS2, and HAS3). Particularly, the HAS2 generates very high molecular mass HAs greater than 2×10^6^ Da whereas Has1 and Has3 generate HA with a mass of 2×10^5^ Da to 2×10^6^ Da. Lung fibroblast and alveolar epithelial cell are major types of lung cells that produce HA, which are subsequently cleaved and released into the pericellular and extracellular matrices. HA is found predominantly in the high molecular weight form in excess of 1000 kDA. The turnover of HA is partly facilitated by hyaluronidases (Hyals), CD44-mediated cellular uptake, fibroblasts, and macrophages then the degradation by Hyal1 and Hyal2. Besides enzyme-mediated degradation, HA is also fragmented by the reactive oxygen species (ROS) that are produced from neutrophils (Lee-Sayer et al., 2015[23]). HA functions as a scaffold in ECM where it contributes to the thickness of the EG in normal condition. Furthermore, it is responsible for the permeable selectiveness and is involved in mechanosensory effect of EG at the vascular vessels upon blood folding. Moreover, HA promotes the activity on the survival along with self-renewal of alveolar macrophages and type II alveolar epithelial cells, particularly, the high molecular weight HA (Chanmee et al., 2015[6]; Zoller, 2015[42]).

## HA Storm during COVID-19 Onset

**Figure 1:**
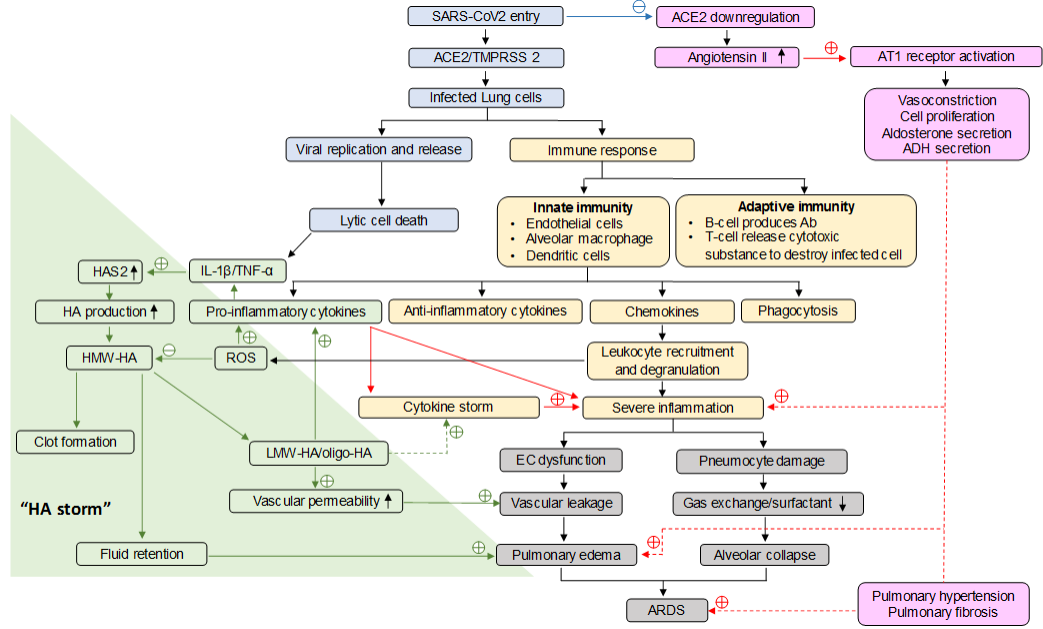
A schematic diagram of SARS-CoV-2 infection shows the involvement of HA synthase, HA storm, cytokine storm, endothelial and immune cells.

Upon infection with SARS-CoV2, a cytokine storm is initiated and causes aggressive inflammatory response leading to the release of a large amount of pro-inflammatory cytokines. Three of the most important pro-inflammatory cytokines of the innate immune response are IL-1β, TNF-α, and IL-6. Therefore, high molecular weight hyaluronan (HMW-HA) production will be dramatically increased due to the HAS2 overexpression which is upregulated by IL-1β and TNF-α. These overproduced and accumulated HMW-HA can absorb high amount of water molecules due to their hygroscopic properties. From the CT scan, white patches containing fluid known as “ground glass” were presented in the lung of COVID-19 patients in accordance with the clear jelly liquid found in lung autopsies (Wang et al., 2020[35]; Xu et al., 2020[39]). It is highly possible that HA is one of the leading factors associated with the lung edema leading up to ARDS. The HA signaling can also vary according to its molecular size and cell types. In the severe lung inflammation, the neutrophils response to eliminate infected cells by producing the ROS that can break down the HMW-HA to the smaller fragments including LMW-HA (low molecular weight hyaluronan) and oligo-HA. This could further enhance the effect of the cytokine storm by stimulating the cytokine release from immune and pulmonary cells. Such event would amplify the inflammation and establishing a feedback loop called "HA storm" leading to increased severity as well as poor prognosis of the patients (Figure 1(Fig. 1)). 

SARS-CoV-2 infection could activate both innate and adaptive immune responses, which may lead to further accumulation of immune cells in the lungs, overproduction of pro-inflammatory cytokines, chemokines, and eventually engender the cytokine storm and severe inflammation. Endothelial cells and pneumocytes were damaged by inflammation process followed by vascular leakage and decreased lung surfactant, respectively. This enhances further pulmonary edema and alveolar collapse leading to the ARDS. Lastly, SARS-CoV-2 infection markedly down-regulated the ACE2 expression in the lungs. Angiotensin II is the most active end product of the RAAS system and acts mainly via angiotensin 1 (AT1). This activation brings about vasoconstriction, cell proliferation, and hormone secretion. This results in an excessive activation of RAAS and exacerbation of ARDS progression.

Although the concise mechanism of HA in COVID-19 remains poorly understood. There are several studies supported that HA is correlated with ARDS (Hallgren et al., 1989[16]; Modig and Hallgren, 1989[27]; Singleton and Lennon, 2011[31]; Uchakina et al., 2013[34]). The levels of HA in bronchoalveolar lavage fluid and serum have been shown to increase in severe adult ARDS patients (Hallgren et al., 1989[16]; Modig and Hallgren, 1989[27]). Alveolar HA appears to be primarily associated with respiratory organ dysfunction rather than systemic organ dysfunction (Esposito et al., 2017[14]).

Most of the severe COVID-19 patients end up with ARDS which possibly imply the involvement of HA in the ARDS mechanism. Remarkably, the levels of serum HA in COVID-19 patients can be used to clearly distinguish critical patients from the mild group (Ding et al., 2020[10]). This may be potentially used as an early indicator for poor prognosis of COVID-19. The over-activation of HAS2 during inflammation causes the accumulation of HMW-HA, which is known for its anti-inflammatory properties. Particularly, this promotes vascular integrity and controls the cellular inflammatory responses. During the severe inflammation, the glycocalyx is shed and the HMW-HA is released and binds with fibrin and fibrinogen to increase clot formation (LeBoeuf et al., 1987[22]). It has been reported that HMW-HA inhibits activity of the extracellular serine protease to protect the degradation of ECM and glycocalyx. HMW-HA also reduces the vascular permeability via the signaling pathways associated with the formation of the cortical layer of actin microfilaments to form dense intercellular contacts. Conversely, LMW-HA increases the vascular permeability by inducing the activation of the protease-activated receptor (PAR) of endothelial cells thereby causing the disruption of intercellular contacts (Ziganshina et al., 2016[41]). LMW-HA, or HA fragments that resulted from the degradation of the intact HMW-HA, have been shown to act as DAMPs, which can mediate and perpetuate an immune response. Fragmented HA is capable of signaling cellular responses through the specific receptors including CD44 and toll-like receptors (TLR) 2 and 4 to potentiate inflammatory response (Lee-Sayer et al., 2015[23]). Moreover, the elevation of HA concentration has been reported to cause hepatic dysfunction thereby leading to decreased hepatic clearance (Wyatt et al., 2002[38]).

## Potential Treatment for HA Storm

**Figure 2:**
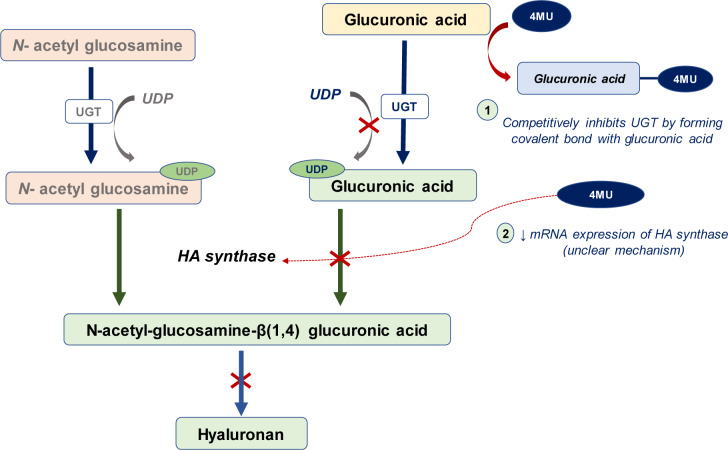
Possible inhibitory mechanisms of 4-methylumbelliferine (4MU) on HA synthesis. 1: 4MU acts as a competitive inhibitor of UGT (an enzyme required for transfer of UDP to glucuronic acid to form precursors of hyaluronan synthesis). 4MU acts in the initial step of HA synthesis by forming a covalent bond with the glucuronic acid, as a result, a substrate for HA synthase (UDP-glucuronic acid) is decreased leading to an inhibition of HA production). 2: 4MU decreases mRNA expression of HA synthase (an enzyme required for the late step of HA synthesis). However, the underlying mechanism of the decreased mRNA expression is still unclear.

The removal of excessive HA in COVID-19 patients would be advantageous to prevent the severity of clinical onsets. At the present time, there are two alternative approaches for eliminating HA. The first approach is to inhibit the HA synthesis via the use of 4-methylumbelliferone (4MU). Particularly, 4MU is a derivative of coumarin that has been used in preventive medicine for prevention of cardiovascular disease owing to its anticoagulatory mechanism. Coumarin hydroxylated at position 7 is known as umbelliferone which is a class of natural product found in many plants. 4MU is a competitive substrate inhibitor for UDP-glucurosyltransferase (UGT), an enzyme synthesis precursor for HA synthesis. As a consequence, the HA synthesis is reduced (Nagy et al., 2015[28]). Additionally, 4MU reduces the HAS mRNA expression of the cells (Kultti et al., 2009[21]) (Figure 2(Fig. 2)). This drug not only inhibits HA synthesis but also reduces the inflammatory cytokine level as demonstrated in mouse infectious models (McKallip et al., 2015[25], 2013[26]). Therefore, 4MU is a promising potential candidate for decreasing HA level in lungs that may improve disease prognosis in severe COVID-19 patients. 

The second approach, the reduction of HMW-HA is mediated by degradative activity of hyaluronidase. The SARS-CoV2 infection causes accumulation of HA, which is similar to the severe case of influenza (Bell et al., 2019[3]). Intranasal administration of exogenous hyaluronidase was reported to sufficiently reduce the level of lung hyaluronan content, thereby restore the lung function in the influenza mouse model (Bell et al., 2019[3]). During inflammation, HA can be exclusively modified by the heavy chain of the serum protein inter-α-inhibitor. This covalently modification of HA is mediated by the induction of the secreted enzyme namely TNFα-stimulated gene-6 (TSG-6). An improvement in the lung function may be attributed to the liberation of I inter-α-inhibitor heavy chains from hyaluronan. Such observation has been observed during the hyaluronidase treatment with inter-α-inhibitor heavy chains·HA complexes. Serious drawbacks should be carefully considered on the breakdown of HMW-HA to LMW-HA which can trigger more cytokine release from the immune cells. However, the treatments with 4MU and hyaluronidase have also been suggested for improving survival rate of severe COVID-19 patients (Shi et al., 2020[30]).

## Conclusion

SARS-CoV-2 infection or COVID-19 has resulted in a devastating pandemic outbreak. The infection primarily affects the lungs, develops ARDS, causes a systemic intricacy leading to multi-organ failure. HA is one of the key factors which incites the severity of COVID-19. Until now, the increasing numbers of the infected people and deaths are still uncontrolled. Thus, the unraveled roles of HA in the severity of SARS-CoV-2 infection may beneficially provide the better clinical management before availability of the vaccine.

## Notes

^1^ **DAMP:** Damage-associated molecular patterns (DAMPs) are intracellular or extracellular molecules releasing from damaged or life-threatening dying cells. When they are relocated into the extracellular environment, they activate the innate immune system by interacting with pattern recognition receptors (PRRs). These DAMPs are recognized by macrophages, and inflammatory responses are triggered by different pathways, including TLRs and inflammasomes (Roh and Sohn, 2018[29]). DAMPs can be classified by their original sources including extracellular proteins (such as biglycan, fibrinogen and LMW-HA) and intracellular compartments (such as high-mobility group box 1 (HMGB1), S100 proteins, and heat shock proteins, HSPs). DAMPs considered to have a pathogenic role in inflammatory diseases.

^2^ **HMGB1:** High mobility group box 1 protein (HMGB1) is a non-histone chromatin protein that is constitutively expressed in almost all of the cell types and is passively released following the traumatic cell death. It is known to induce inflammation via an activation of NF-kB pathway by binding to TLR, TLR4, TLR9, and RAGE (Andersson and Tracey, 2011[2]). HMGB1 induces dendritic cell maturation via an upregulation of CD80, CD83, CD86 and CD11c (Dumitriu et al., 2005[13]). It also increases the productions of pro-inflammatory cytokines including IL-1, TNF-α, IL-6 and IL-8. 

^3^ **RAGE:** Receptor for advanced glycation end products (RAGE) is a multifunctional transmembrane protein belonging to the superfamily of immunoglobulin. It is engaged in many crucial processes, including inflammation, proliferation, apoptosis, autophagy, and migration. Dysregulation of RAGE and its ligands could lead to the development of numerous pathological conditions (Chuah et al., 2013[8]).

## Conflict of interest

The authors declare that they have no conflict of interest.
